# Regarding: Koilocytes indicate a role for human papilloma virus in breast cancer

**DOI:** 10.1038/sj.bjc.6605549

**Published:** 2010-02-02

**Authors:** R E Sandstrom

**Affiliations:** 1 Lower Columbia Pathologists, Longview, WA, USA

**Sir,**

I welcome the contributions of Heng *et al* and Lawson *et al* in the October 2009 issue of the *British Journal of Cancer*. I would like to comment on the article submitted by Lawson *et al*, ‘Koilocytes indicate a role for human papilloma virus in breast cancer’. The authors cite an earlier article in which I was a participating author and refer to the koilocytes identified in our article as ‘putative (supposed) HPV-associated koilocytes’. The concept of koilocytosis was initially and arguably still remains a morphological construct (Reid *et al*, 1982). In the early 1990s, based on a systematic and careful review of routine breast cancer specimens, which included areolar and nipple tissue, Buck and I submitted, for publication, a select series of breast cancer cases with koilocytosis in the nipple and areola. The initial attempts at publication were rejected because of an absence of molecular confirmation of the presence of human papilloma virus (HPV). Subsequently, with de Villiers (Buck *et al*, 2001), we reported a limited series of areola and nipple specimens with histological evidence of koilocytosis confirmed by polymerase chain reaction (PCR). In a larger series, joined by zur Hausen (de Villiers *et al*, 2005), we published a series of cases including both nipple and areolar specimens and associated breast cancer tissue. In that report, both PCR and *in situ* hybridization were used to confirm the presence of the viral DNA in koilocytes. We demonstrated the co-localization by *in situ* hybridization with histological evidence of koilocytosis. Our confirmatory methods were in essence identical to those used by Lawson *et al.*

Recent identification of HPV by PCR in breast milk, ductal lavage and colostrum specimens is not cited by Lawson *et al* (Sarkola *et al*, 2008; Cazzaniga *et al*, 2009). Human papilloma virus was detected in 4 and 14% of non-selected breast milk specimens. Although the authors identified low numbers of positive HPV samples, the time-specific incidence of HPV positivity is of the same order of magnitude as encountered in cervical specimens of sexually active women and, to my mind, implies a relatively high aggregate lifetime likelihood of HPV colonization of the nipple and/or areola. Cazzaniga *et al* have argued that scraping the superficial layers of the nipple decreased the number of positive samples. However, the argument can be made that this may reflect the natural history of HPV in that virus accumulates through the course of keratinizing cell maturation from the basal layer to the superficial layer. It has been documented that sampling from the surface of skin tumours after striping the biopsy with an adhesive tape decreases the likelihood of isolating the virus by PCR (Forslund *et al*, 2004). The presence of the virus in breast milk specimens supports the likelihood that HPV infects the nipple and areolar tissue and indirectly supports the concept of HPV-specific koilocytosis at these sites. I hope I will be allowed some latitude for speculation in this regard. There is the possibility that HPV transmission in the neonatal period in breast milk or colostrum through immune modulation and oral induced tolerance (Strobe *et al*, 2001; Lawrence and Lawrence, 2004) may modify host immune response to the virus encountered later in life. This speculative construct may further complicate and enrich the pathophysiological mechanism that Buck *et al* initially postulated that HPV infection as evidenced by koilocytosis affecting the nipple, areola and duct structures may be related to the induction of neoplasia in breast tissue by retroductular spread of the virus.
